# Supplementary material for: Eco-sustainable magnetoresistive sensors towards disposable magnetoelectronics
Source: Nat Commun. 2026 Mar 27;17:3034. doi: 10.1038/s41467-026-71077-9 (PMC13036069; doi:10.1038/s41467-026-71077-9)
Supplement: Supplementary file 1 — Supplementary Information [file 41467_2026_71077_MOESM1_ESM.pdf]

Eco-sustainable magnetoresistive sensors towards disposable magnetoelectronics

Lin Guo[1], Rui Xu[1*], Proloy Taran Das[1], Eduardo Sergio Oliveros-Mata[1], Xuan Peng[2], Oleksandr V. Pylypovskyi[1], René Hübner[1], Fabian Ganss[1], Xiaotao Wang[1], Yi Li[1], Sebastian Gepp[3], Yevhen Zabila[1], Xilai Bao[4], Shengbin Li[4], Qihao Zhang[1], Igor Veremchuk[1], Željko Janićijević[2], Larysa Baraban[2], Clemens Voigt[5], Sindy Mosch[5], Oliver Gutfleisch[6], Run-Wei Li[4,7] & Denys Makarov[1*]

[1]Helmholtz-Zentrum Dresden-Rossendorf e.V., Institute of Ion Beam Physics and Materials Research, Bautzner Landstrasse 400, 01328 Dresden, Germany

[2]Helmholtz-Zentrum Dresden-Rossendorf e. V., Institute of Radiopharmaceutical Cancer Research, Bautzner Landstrasse 400, 01328 Dresden, Germany

[3]Freudenberg Siebdruck GmbH, Am Feld 4, 01257 Dresden, Germany

[4]CAS Key Laboratory of Magnetic Materials and Devices, Ningbo Institute of Materials Technology and Engineering, Chinese Academy of Sciences, 315201 Ningbo, P. R. China

[5]Fraunhofer Institute for Ceramic Technologies and Systems IKTS, Winterbergstrasse 28, 01277 Dresden, Germany

[6]Institute of Materials Science, Technical University of Darmstadt, 64287 Darmstadt, Germany

[7]Eastern Institute of Technology, 315200 Ningbo, P. R. China

E-mails: r.xu@hzdr.de (R.X.), d.makarov@hzdr.de (D.M.)

Supplementary Table 1. Comparison of the fabrication, biodegradability, biocompatibility and low field (10 mT) magnetoresistive performance of printed MR sensors. (*) marks values that were estimated from graphs in the indicated publications.

Type	Functional element	Binder	MR (10 mT)	Sensitivity	Figure of Merit	Green fabrication	Large-scale fabrication	Recyclablilty	Biodegra-dability	Biocompatibility	Ref.
GMR	$[Co/Cu]_{50}$ stack microflakes	Polymethyl methacrylate (PMMA)	*0.3 %	*0.06 T^{-1}	*0.68 T^{-2}	No<br>Methyl isobutyl ketone<br>Sputtering	No	No	No	No	1
	$[Co/Cu]_{50}$ stack microflakes	Polyepichlorohydrin (PECH)	*0.7 %	0.93 T^{-1}	*7.15 T^{-2}	No<br>Acetone<br>Sputtering	No	No	No	No	2
	$[Py/Cu]_{30}$ stack microflakes	Poly(styrene-butadiene-styrene) (SBS)	*1.5 %	3 T^{-1}	3409 T^{-2}	No<br>Xylol<br>Sputtering	No	No	No	No	3
	$[Co/Cu]_{30}$ stack microflakes	Polyepichlorohydrin (PECH)	*0.2 %	*0.78 T^{-1}	*3.55 T^{-2}	No<br>Ethyl acetate<br>Sputtering	Yes	No	No	No	4
	$[Co/Cu]_{50}$ stack microflakes	Polyepichlorohydrin (PECH)	1.4 %	1.8 T^{-1}	*120 T^{-1}	No<br>Acetone<br>Sputtering	No	Yes	No	No	5
	FeCoNi/Cu nanowires	Polydimethylsiloxane (PDMS)	*0.25 %	*0.55 T^{-1}	*2.43 T^{-2}	No<br>NaOH, HNO_3<br>$Ni(SO_3NH_2)$	No	No	No	No	6

**AMR**	Bilayer [Ta (5 nm)/Py (100 nm)] microflakes	Poly(styrene-butadiene-styrene) (SBS)	*0.1 %	1.9 T^{-1}	*380 T^{-2}	No<br>Xylol<br>Sputtering	No	No	No	No	7
	NiCo microparticles	Polyepichlorohydrin (PECH)	0.33 %	0.9 T^{-1}	*180 T^{-2}	No<br>Acetone	No	Yes	No	No	5
	NiFe microparticles	Polyborosiloxane (PBS)	*1 %	35.7 T^{-1}	4.1×10^5 T^{-2}	No<br>n-Hexane<br>AMF Treating	No	No	No	No	8
	NiFe nanowires	Polydimethylsiloxane (PDMS)	0.03%	0.086 T^{-1}	0.3 T^{-2}	No<br>HCl, NaOH	No	No	No	No	9
**LMR**	Bi microparticles	Butylmethacrylate	*0.2 %	0.35 T^{-1}	0.07 T^{-2}	No<br>Toluene<br>Laser	No	No	No	No	10
**TMR**	Fe-hexadecylamine ligand nanoparticles	Hexadecylamine ligand	*0.01 %	*0.023 T^{-1}	*0.09 T^{-2}	No<br>Toluene<br>Mesitylene	No	No	No	No	11
	FeCo nanoparticles	Hexadecylamine (HDA) Hexadecylammonium (HCL)	*0.1 %	*0.066T^{-1}	*0.44 T^{-2}	No<br>Mesitylene<br>Toluene	No	No	No	No	12

**GB-MR**	Fe/Fe_3O_4 core-shell microparticles	Sodium carboxymethyl cellulose (NaCMC)	1.7 %	3.93 T^{-1}	3046 T^{-2}	Yes Water	Yes	Yes	Yes	Yes	This work

Supplementary Table 2. Comparison of the electrical and low field (10 mT) magnetoresistive performance of Fe-based MR sensors. (*) marks values that were estimated from graphs in the indicated publications.

Type	Materials	Particles size	Method of fabrication	MR	MR at 10 mT	Sensitivity	FoM	Resistivity	Ref.
**Single crystal**	Fe epitaxial thin film	Single crystal thin film	MBE	0.08% [001] direction<br>0.35% [110] direction<br>0.51% [111] direction<br>(at 0.2 T)	\	\	\	2.1×10^{-7} Ω·m	13
	Fe single crystal	Single crystal whiskers	Hydrogen reduction of $FeCl_2$	0.2% [100] direction<br>0.4% [111] direction<br>(at 0.4 T)	\	\	\	\	14
	Fe single crystal	Single crystal whiskers	Hydrogen reduction of $FeCl_2$	0.12% [100] direction<br>0.4% [111] direction<br>(at 0.2 T)	\	\	\	\	15
**Core shell particles**	Core shell Fe nanoparticles Hexadecylamine and hexadecylammonium	9 nm	Dielectrophoresis	0.75 % (at 3 T)	*<0.1%	*0.013 T^{-1}	*0.28 T^{-2}	$>1\times10^{5}$ Ω·m	11
	Core shell Fe nanoparticles Hexadecylamine and palmitic acid	11 nm	Dielectrophoresis	0.6% (at 10 T)	*<0.1%	*0.0018 T^{-1}	*0.013 T^{-2}	$>1\times10^{5}$ Ω·m	11
	Core-shell Fe nanoparticles/MgO	150 nm	Cold pressing	0.37 % (at 4 T)	*<0.1%	*0.0035 T^{-1}	*0.013 T^{-2}	\	16
**Nanoparticles with carbonized polymer**	Fe nanoparticles with carbonized polyurethane	20 nm	Sintering (5% H_2 with Ar)	7.3% (at 9 T)	*<0.1%	*0.022 T^{-1}	*0.066 T^{-2}	\	17

	Fe nanoparticles with carbonized vinyl ester resin	20 nm	Sintering (5% H_2 with Ar)	8.3 % (at 9 T)	*<0.1%	*0.065 T^{-1}	*0.24 T^{-2}	1.5×10^{-2} Ω·m	18
	Fe nanoparticles with carbonized polyacrylonitrile	20 nm	Solvent extracted and sintering (5% H_2 with Ar)	5% (at 9 T)	*<0.1%	*0.06 T^{-1}	*0.66 T^{-2}	\	19
**This work**	Fe polycrystalline film	Polycrystalline film (1.5 µm thickness)	Sputtering	0.12% (at 0.1 T)	0.08%	0.47 T^{-1}	940 T^{-2}	$1*10^{-6}$ Ω·m	This work
	Fe microparticles with NaCMC binder (acid wash)	3 µm	Printing	0.08% (at 0.1T)	0.04%	0.098 T^{-1}	32.6 T^{-2}	1×10^{-3} Ω·m	This work
	Fe/Fe_3O_4 core-shell microparticles with NaCMC binder	3 µm	Printing	3.1 % (at 0.1 T)	1.7%	3.93 T^{-1}	3046 T^{-2}	6×10^{-1} Ω·m	This work

Supplementary Table 3. Comparison of the electrical and low field (10 mT) MR performance of Fe_3O_4 based MR sensors. (*) marks values that were estimated from graphs in the indicated publications.

Type	Materials	Particles size	Method of fabrication	Magnetoresistance	MR at 10 mT	Sensitivity	FoM	Resistivity	Ref.
**Polycrystalline film**	Fe_3O_4 thin film	640 nm (thickness)	magnetron sputtering	4.4% (at 9 T)	*<0.1%	*0.013 T^{-1}	0.02 T^{-2}	2×10^{-3} Ω·m	20
	Fe_3O_4 thin film	330 nm (thickness)	magnetron sputtering	7.4% (at 5 T)	*<0.1%	*0.03 T^{-1}	*0.05 T^{-2}	1.8×10^{-3} Ω·m	21
	Fe_3O_4 thin film	300 nm (thickness)	reactive evaporation	5.4% (at 6 T)	*<0.1%	*0.03 T^{-1}	*0.35 T^{-2}	2.2×10^{-3} Ω·m	22
**Particles**	Fe_3O_4 nanoparticles	33 nm	cold pressing	3.6% (at 2 T)	*0.1%	*0.1 T^{-1}	*3.7 T^{-2}	1.6×10^{-2} Ω·m	23
	Fe_3O_4 microparticles	1 µm	cold pressing	1.2% (at 0.5 T)	*<0.1%	*0.12 T^{-1}	*2.31 T^{-2}	5×10^{-1} Ω·m	24
	Fe_3O_4 microparticles with NaCMC binder	5 µm	printing	1.9% (0.5 T)	0.13%	0.21 T^{-1}	5.53 T^{-2}	1×10^{2} Ω·m	This work

**Particles with organic ligand**	Fe_3O_4 nanoparticles with acetic acid ligands	450-650 nm	casting	11% (at 0.3 T)	*2%	*4.68 T^{-1}	*669 T^{-2}	1×10^{4} Ω·m	25
	Fe_3O_4 nanoparticles with octadecanoic acid ligands	9 nm	casting	21% (at 9 T)	*0.8%	*0.47 T^{-1}	*5.87 T^{-2}	2×10^{6} Ω·m	26
	Fe_3O_4 nanoparticles with TTF-COO- ligands	5.7 nm	cold pressing	5% (0.6 T)	*0.1%	*0.14 T^{-1}	*1.55 T^{-2}	2×10^{5} Ω·m	27
	Fe_3O_4 nanoparticles with tetrathiafulvalene-fused carboxylic ligands	5.7 nm	cold pressing	1.4% (at 0.6 T)	*<0.1%	*0.054 T^{-1}	*0.85 T^{-2}	6×10^{3} Ω·m	28
	Fe_3O_4 nanoparticles with oleylamine ligands	7.0 nm	cold pressing	8.5% (at 0.45 T)	*0.2%	*0.31 T^{-1}	*4.56 T^{-2}	2×10^{4} Ω·m	29
**Fe/Fe_3O_4 core shell microparticles**	Fe/Fe_3O_4 core shell microparticles with NaCMC binder	3 µm	printing	3.1% (at 0.1 T)	1.7%	3.93 T^{-1}	3046 T^{-2}	6×10^{-1} Ω·m	This work

Supplementary Table 4. Device-to-device and batch-to-batch reproducibility of printed Fe/Fe_3O_4 sensors. Statistical summary of key performance parameters measured for three independently fabricated batches (Batch 1-3, n = 10 devices per batch; total n = 30): magnetoresistance at 10 mT (MR@10 mT), saturated magnetoresistance at 100 mT (MR@100 mT), and maximum sensitivity (S_{max}). For each batch and for the entire dataset, the mean, standard deviation (SD), and coefficient of variation (CV) are reported.

**Batch Number**	**Sample number**	**MR at 10 mT**	**Saturated MR (at 100 mT)**	**Max Sensitivity (T^{-1})**
**Batch #1**	#1	2.0%	3.2%	4.52
	#2	1.7%	2.8%	3.96
	#3	1.6%	2.9%	3.69
	#4	1.7%	2.9%	3.96
	#5	1.8%	2.9%	4.05
	#6	1.7%	3.1%	3.85
	#7	1.5%	2.9%	3.52
	#8	1.7%	2.9%	3.92
	#9	1.8%	3.0%	3.98
	#10	1.9%	3.4%	4.31
	Mean	1.74%	3.00%	3.98
	SD	0.14%	0.18%	0.33
	CV	8.05%	6.00%	8.29%
**Batch #2**	#1	1.6%	2.9%	3.61
	#2	1.6%	3.1%	3.55
	#3	1.6%	2.8%	3.65
	#4	1.6%	2.9%	3.72
	#5	1.9%	3.0%	4.27
	#6	1.8%	3.0%	4.12
	#7	1.5%	3.0%	3.57
	#8	1.8%	3.1%	4.04
	#9	1.7%	3.0%	3.88
	#10	1.6%	3.0%	3.81
	Mean	1.67%	2.98%	3.82
	SD	0.13%	0.09%	0.25
	CV	7.78%	3.02%	6.54%
**Batch #3**	#1	1.9%	3.1%	4.32
	#2	1.9%	3.1%	4.22
	#3	1.9%	3.1%	4.24
	#4	1.9%	3.2%	4.18
	#5	1.9%	3.2%	4.17
	#6	2.1%	3.4%	4.62
	#7	2.1%	3.4%	4.75
	#8	2.0%	3.4%	4.47
	#9	2.0%	3.0%	4.39
	#10	1.9%	3.2%	4.29
	Mean	1.96%	3.21%	4.37
	SD	0.08%	0.14%	0.19
	CV	4.08%	4.36%	4.35%
**All**	Mean	1.79%	3.06%	4.05
	SD	0.17%	0.18%	0.33
	CV	9.50%	5.88%	8.15%

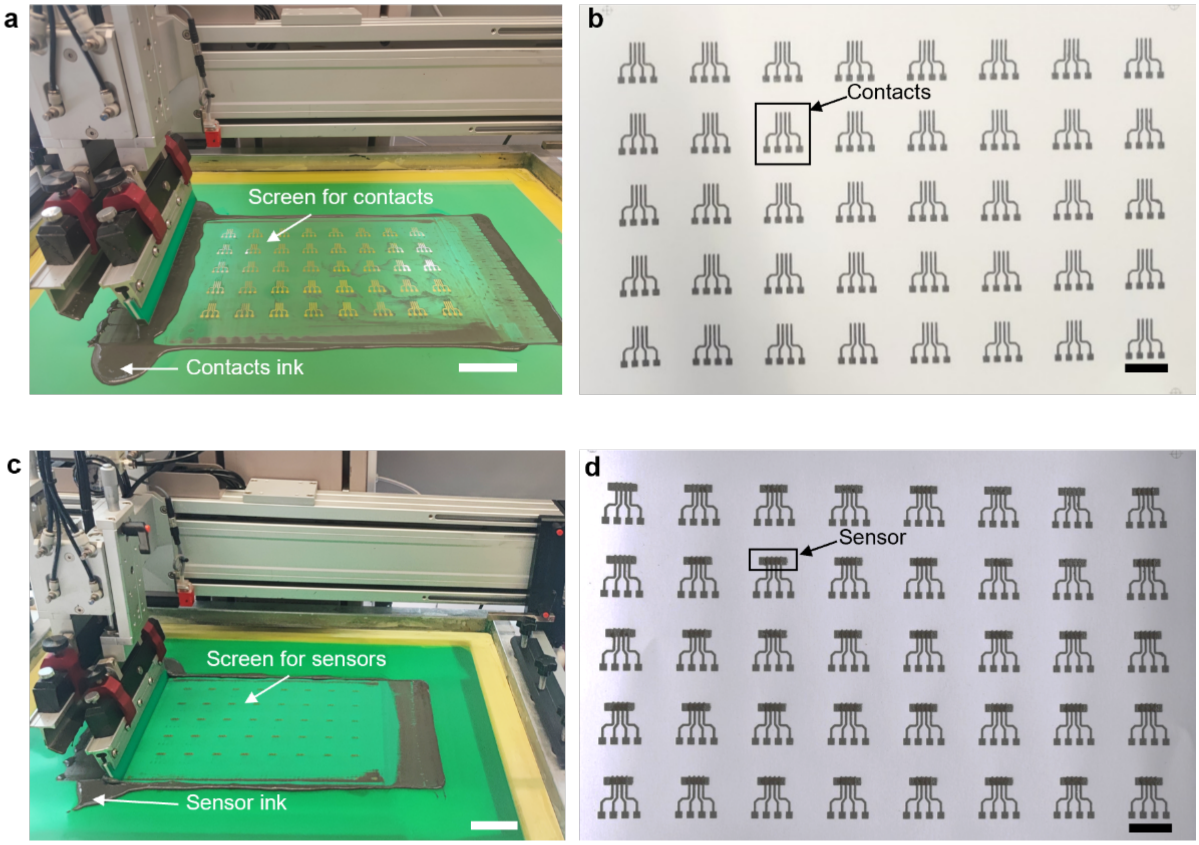

Supplementary Fig. 1. Photograph of the screen-printing process for large scale manufacturing. **a.** Screen printing process of electrode array. Scale bar, 5 cm. **b.** Printed electrode array (5 × 8) on A4 size paper. Scale bar, 2 cm. The electrode ink was composed by Mo microparticles and NaCMC-water solution. **c.** Screen printing process of sensor array. Scale bar, 5 cm. **d.** Printed sensor-electrode array (5 × 8) on A4 size paper. Scale bar, 2 cm. The sensor ink was composed by Fe/Fe_3O_4 core-shell microparticles and NaCMC-water solution.

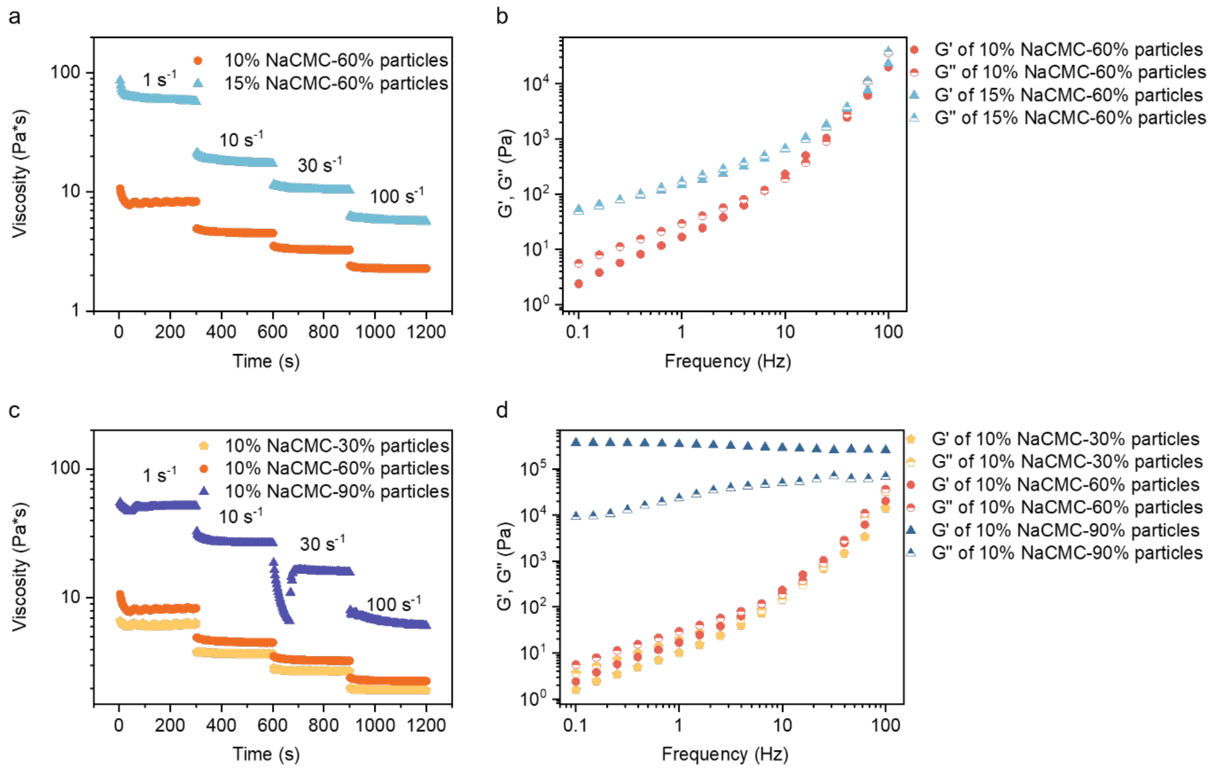

Supplementary Fig. 2. Rheological characterization of NaCMC-Fe/Fe_3O_4 inks for screen printing. **a.** Stepwise steady-shear viscosity of inks containing 60 vol% Fe/Fe_3O_4 particles (in the final dried composite) formulated using NaCMC aqueous binders with different NaCMC concentrations (10 wt% and 15 wt% NaCMC in water). The shear rate was sequentially set to 1, 10, 30, and 100 s^{-1} (values indicated in the panel). **b.** Frequency-dependent storage modulus (G′) and loss modulus (G″) of the inks in panel **a** measured by small-amplitude oscillatory shear. **c.** Stepwise steady-shear viscosity of inks formulated with a 10 wt% NaCMC aqueous binder and different Fe/Fe_3O_4 particles volume fractions (30, 60, and 90 vol% in the final dried composite). **d.** Corresponding oscillatory frequency sweeps of G′ and G″ for the inks in **c**. NaCMC concentration refers to the polymer weight fraction in the aqueous binder solution used for ink preparation; particles content refers to the Fe/Fe_3O_4 particle volume fraction in the composite after solvent evaporation. All measurements were performed at 22°C using a plate-plate geometry (1 mm gap).

Rheological characterization was conducted to evaluate the printability window of NaCMC-Fe/Fe_3O_4 composite inks by varying the NaCMC concentration in the aqueous binder and the Fe/Fe_3O_4 particles volume fraction in the final dried composite. First, viscosity of the ink is in the range that is suitable for screen printing. The stable formulations exhibit pronounced shear-thinning behavior in stepwise steady-shear tests: the viscosity decreases systematically when the shear rate is increased from 1 to 100 s^{-1}, which is desirable for screen printing because the ink can flow readily through the mesh under the squeegee yet recover a higher viscosity at low shear to suppress spreading after printing. Increasing the NaCMC concentration (10 wt% → 15 wt%, at 60 vol% Fe/Fe_3O_4 particles) substantially raises the low-

shear viscosity and the viscoelastic moduli (G′ and G″), indicating a strengthened polymer/particle network that improves the suspension stability and shape retention. In contrast, inks formulated with a 5 wt% NaCMC binder at 60 vol% Fe/Fe_3O_4 particles were not stable (rapid sedimentation and phase separation), evidencing that an insufficient binder concentration fails to provide an adequate yield/structural strength for printing.

At the fixed NaCMC concentration (10 wt%), increasing the Fe/Fe_3O_4 particles loading (30 → 60 → 90 vol%) progressively elevates the viscosity and moduli. The 90 vol% Fe/Fe_3O_4 particles ink shows a markedly solid-like response with high, weakly frequency-dependent G′ and G′ ≫ G″ across the measured range, consistent with a jammed/percolated particle skeleton. While such a structure can aid shape fidelity, it may also impede mesh transfer and increase the risk of screen clogging. By comparison, 10 wt% NaCMC-60 vol% Fe/Fe_3O_4 particles provides a balanced rheological profile-stable dispersion, strong shear-thinning, and stable viscoelasticity—supporting reliable screen printing with good pattern fidelity and minimal slumping.

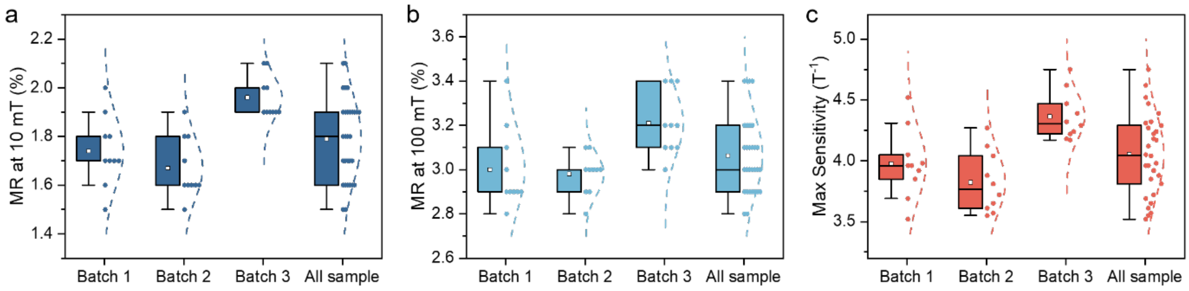

Supplementary Fig. 3. Within-batch and batch-to-batch reproducibility of printed Fe/Fe_3O_4 sensors. a. Magnetoresistance at 10 mT (MR@10 mT) for three independently fabricated batches (Batch 1-3, n = 10 devices per batch) and the entire dataset (all samples, n = 30). **b.** Saturated magnetoresistance at 100 mT (MR@100 mT) for the same samples. **c.** Maximum low-field sensitivity (S_{max}) for the same samples. In all panels, box plots indicate the median (center line), interquartile range (box), and 1.5× interquartile range (IQR) whiskers; individual data points represent measurements from independent samples.

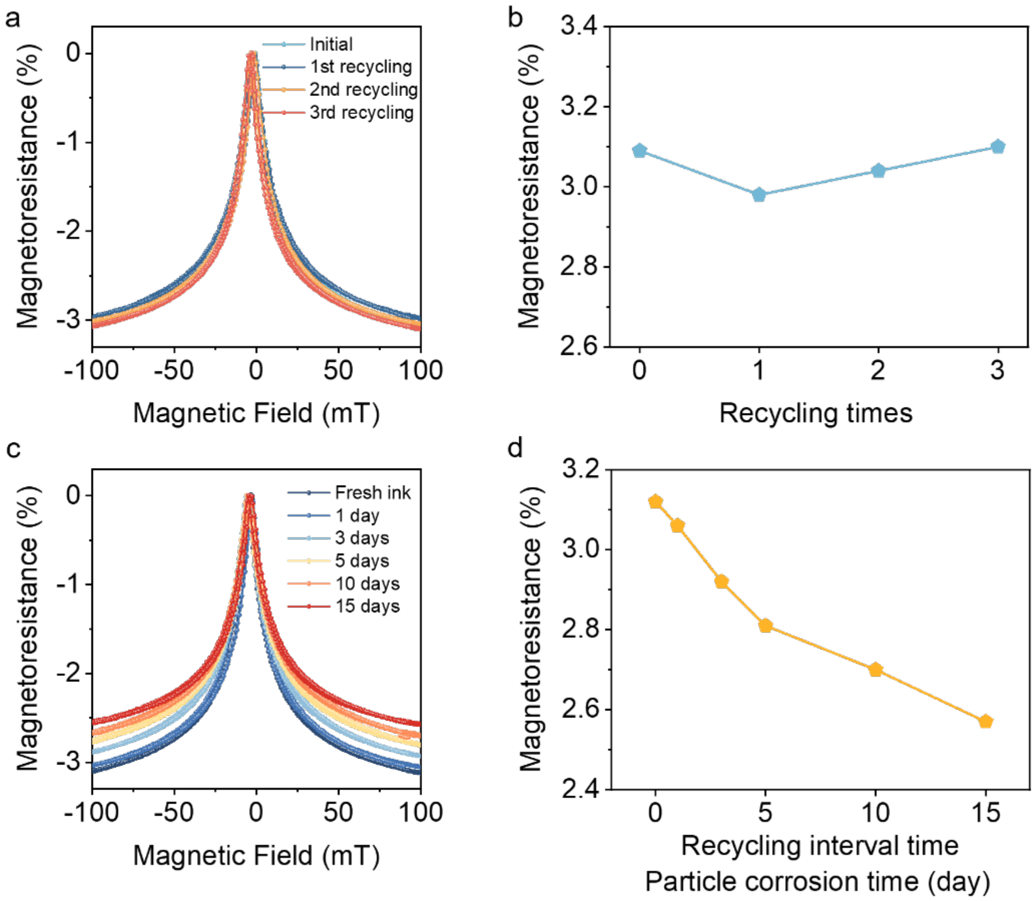

Supplementary Fig. 4. Effect of the recycling procedure on the MR performance of reprinted sensors. a. MR curves of sensors printed with pristine Fe/Fe_3O_4 microparticles (Initial) and with particles recovered after 1st-3rd recycling cycles. In each recycling cycle, the NaCMC binder was dissolved in water and the dispersed Fe/Fe_3O_4 particles were collected magnetically; the particle residence time in water was kept below 1 h. **b.** Corresponding MR ratio at 100 mT as a function of recycling cycle. **c.** MR curves of sensors reprinted using Fe/Fe_3O_4 particles exposed to water for different immersion durations (particle corrosion time) during a single recycling step (1-15 days), showing progressive performance degradation upon prolonged immersion. **d.** Corresponding change of the MR performance as a function of the immersion (corrosion) time.

The recycling of the printed sensors relies on the water solubility of the NaCMC binder. During end-of-life processing, the printed composite disintegrates in water as NaCMC dissolves, releasing Fe/Fe_3O_4 particles into the suspension. Owing to their intrinsic magnetism, the particles can be efficiently collected using a permanent magnet, dried, and re-dispersed in a freshly prepared NaCMC aqueous solution to formulate the ink for reprinting. This water-enabled dissolution-magnetic collection route constitutes a key step enabling closed-loop recyclability without harsh reagents.

Importantly, when the recycling step is performed promptly, the MR performance of reprinted sensors remains essentially unchanged. As shown in panels **a** and **b,** particles recovered through three consecutive recycling cycles, each with a short water exposure time (< 1 h), yield reprinted sensors

whose MR curves nearly overlap with that of the initial device, and the MR ratio at 100 mT stays around 3%. This indicates a good reusability of the Fe/Fe_3O_4 particles under practical, time-efficient recycling conditions.

In contrast, a prolonged water immersion leads to a gradual decline in the MR performance (panels **c** and **d**). When Fe/Fe_3O_4 particles are kept in water for extended durations during a single recycling step, the MR performance gradually decreases, reaching 2.57% after 15 days of immersion, due to the water induced corrosion.

These observations provide practical guidance for both fabrication and recycling. First, because the ink uses water as the solvent and the particles are continuously exposed to an aqueous environment once mixed, prolonged storage can induce ink aging and performance loss. Therefore, the ink should be prepared on demand (i.e., mix NaCMC aqueous solution with Fe/Fe_3O_4 particles shortly before printing). Second, during recycling, the residence time of particles in water should be minimized. Notably, since the performance degradation becomes evident only after multi-day immersion while typical recycling requires less than 1 h per cycle, water-induced corrosion is a relatively slow process in comparison to the practical recycling timescale. Consequently, a reasonable number of recycling loops is expected to have no impact on the MR performance of reprinted sensors, supporting the feasibility of closed-loop reuse of the MR fillers.

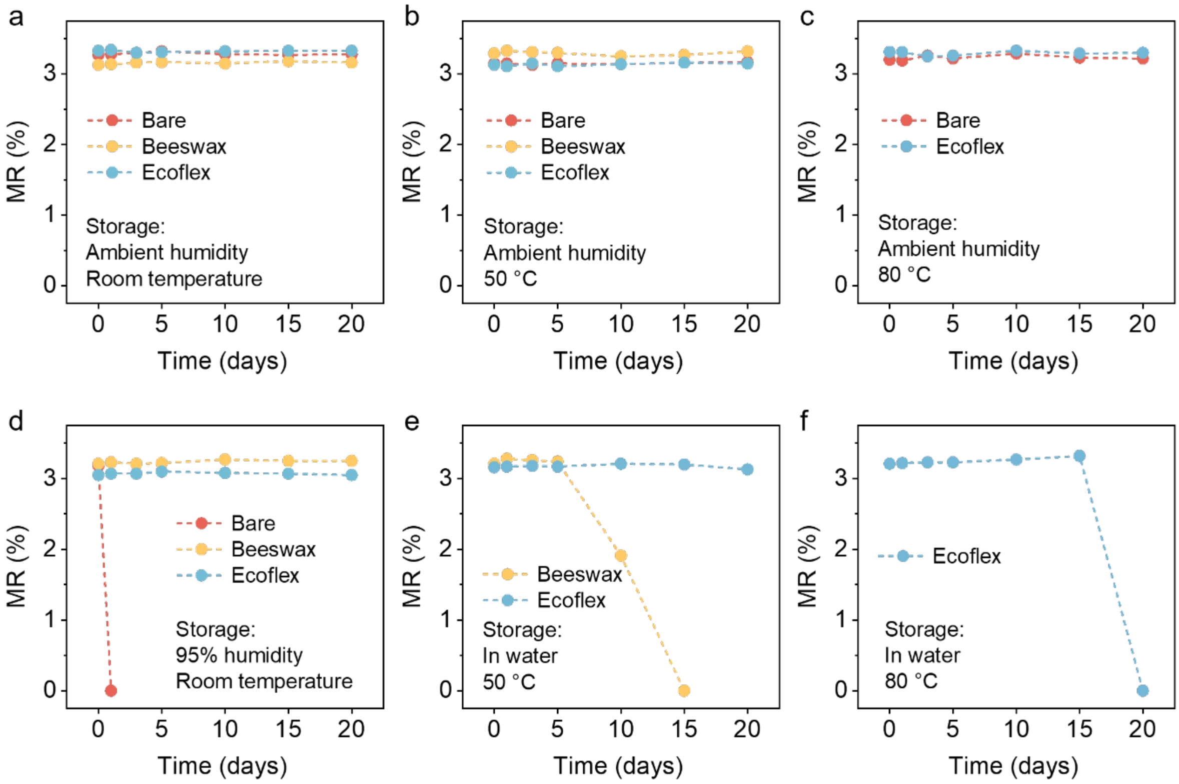

Supplementary Fig. 5. Effect of encapsulation and storage conditions on MR stability of printed Fe/Fe_3O_4-NaCMC sensors. MR ratio (at 100 mT) of printed sensors without encapsulation (Bare) and with beeswax or Ecoflex encapsulation after storage under **a.** ambient humidity at room temperature, **b.** ambient humidity at 50°C, **c.** ambient humidity at 80°C, **d.** 95% relative humidity at room temperature, **e.** immersion in water at 50°C, and **f.** immersion in water at 80°C. Beeswax was not tested at 80°C due to melting. MR was measured at room temperature after the indicated storage time. Data points at 0% correspond to the loss of electrical conduction (device failure).

The printed Fe/Fe_3O_4-NaCMC composite is intrinsically moisture-sensitive because the NaCMC matrix is hydrophilic and water-soluble; moisture uptake can swell/soften the binder and disrupt the particle percolation network, leading to a rapid increase in resistance and eventual loss of conductivity. The sensors remain stable for at least 20 days under ambient humidity even when continuously stored at up to 80°C, whereas unencapsulated devices fail rapidly at 95% relative humidity (RH). The encapsulation effectively delays the moisture ingress and thus expands the operational window: beeswax and Ecoflex both protect the device in high humidity at room temperature, while under water immersion the lifetime becomes temperature and encapsulation dependent. Beeswax provides only short-term protection at elevated temperatures (accelerated degradation in warm water and melting at ~80°C), whereas Ecoflex offers the widest window (stable for ≥20 days in water at 50°C and ~15 days at 80°C). Together with the long-term room-temperature immersion results in the Fig. 2c (main text), these data highlight that the service lifetime can be application-specifically tuned by selecting an appropriate biodegradable encapsulation strategy.

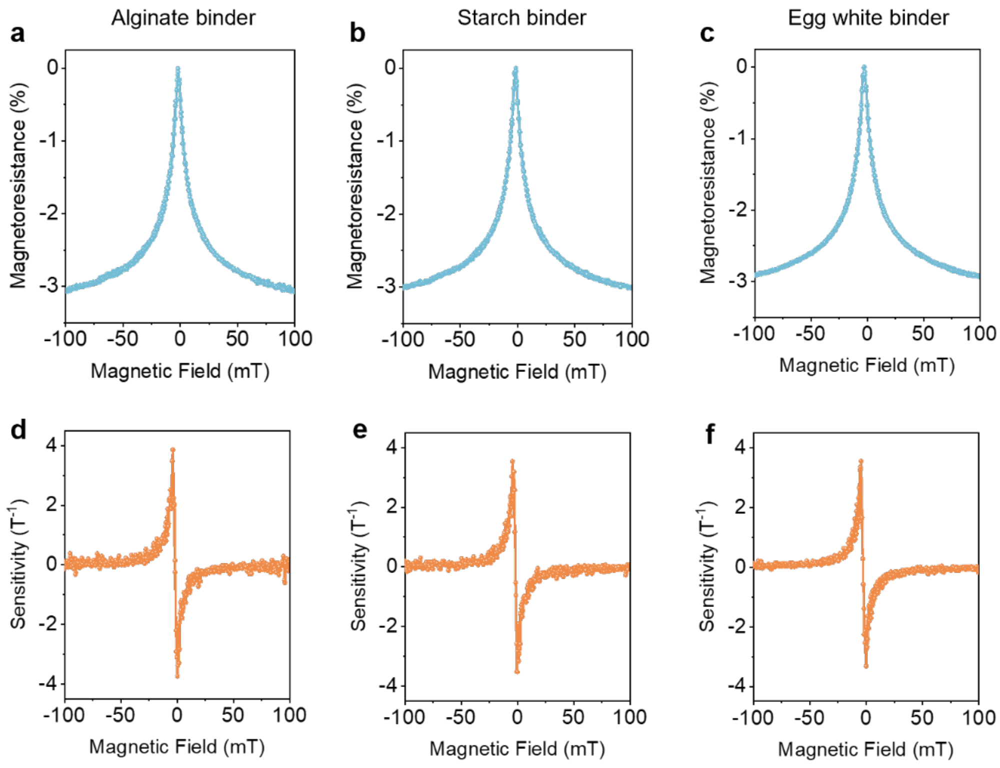

Supplementary Fig. 6. Characterization of printed MR sensors fabricated by different binders. a-c. Magnetoresistance and **d-f.** magnetoresistive sensitivity of the sensors fabricated by **a, d.** alginate, **b, e.** starch, **c, f.** egg white.

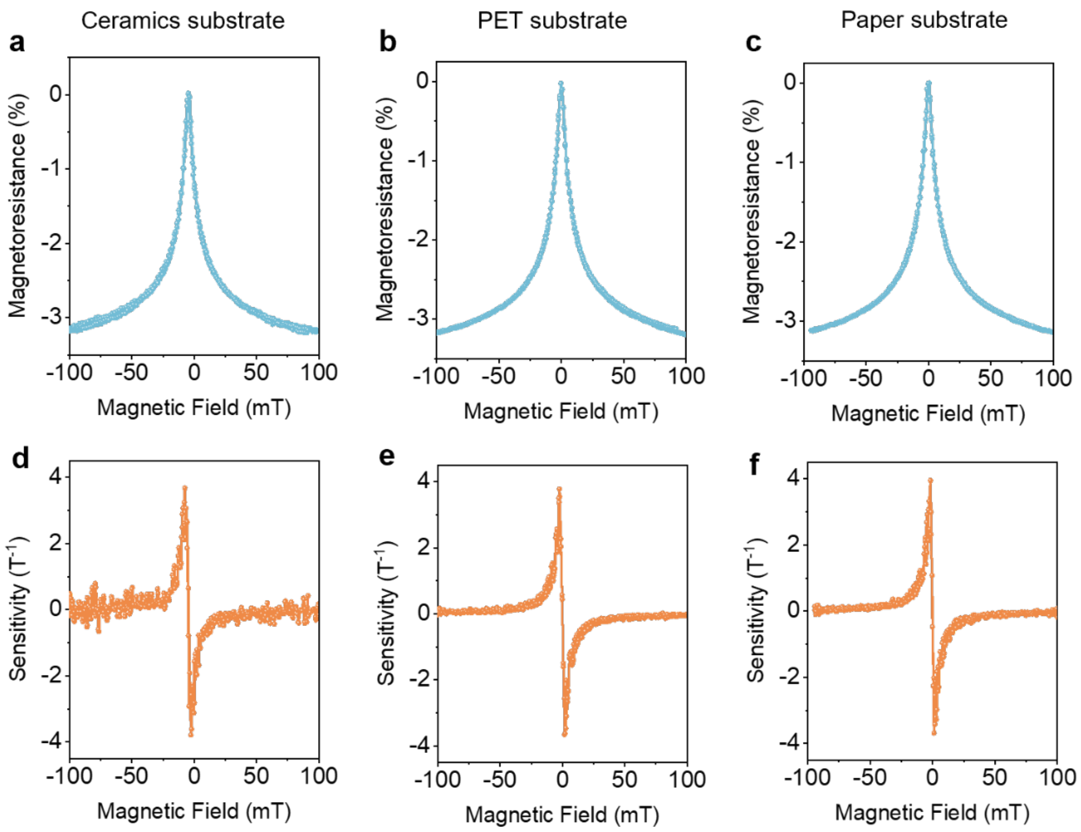

Supplementary Fig. 7. Characterization of printed MR sensors fabricated on different conventional substrates. **a-c.** Magnetoresistance and **d-f.** magnetoresistive sensitivity of the sensors fabricated on **a, d.** ceramics, **b, e.** PET, **c, f.** paper.

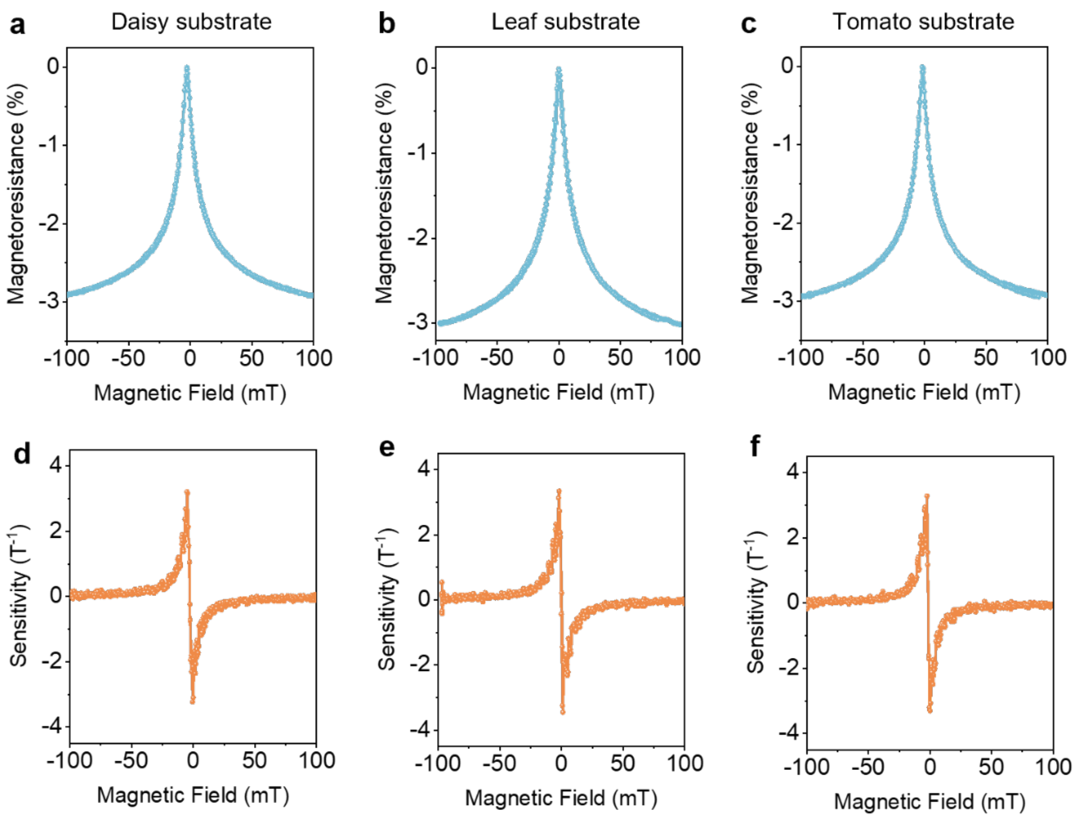

Supplementary Fig. 8. Characterization of printed MR sensors fabricated on different bio-based substrates. **a-c.** Magnetoresistance and **d-f.** magnetoresistive sensitivity of the sensors fabricated on **a, d.** daisy petal, **b, e.** leaf, **c, f.** tomato.

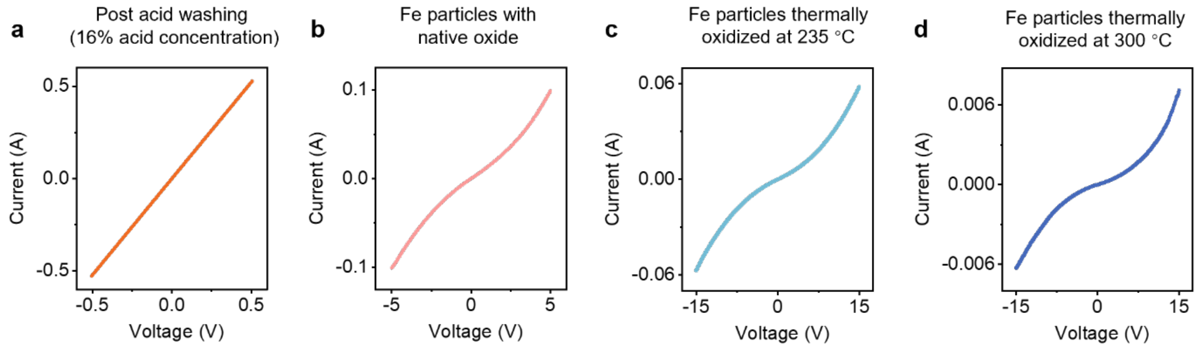

Supplementary Fig. 9. Room temperature current-voltage (I-V) characteristics. **a.** printed sample post treated by acid washing (16% acid concentration) **b.** sample printed with commercial Fe particles with native oxide **c.** sample printed with Fe particles thermally oxidized at 235°C **d.** sample printed with Fe particles thermally oxidized at 300°C. The thermal oxidation was performed under 0.5 mbar vacuum. The sample post-treated by acid washing (reducing oxide) exhibit a linear I-V behavior, indicating the metallic transport behavior. The samples with oxide shell show the nonlinear I-V behavior. It's explained by variable-range hopping (VRH) transport in disordered, granular powder systems.

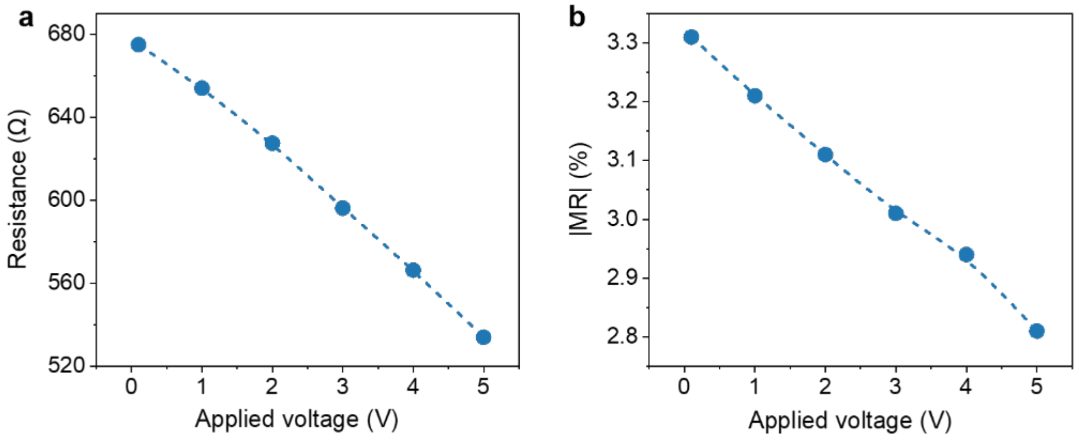

Supplementary Fig. 10. a. DC resistance of Fe/Fe_3O_4 core-shell particles decreases with increasing applied voltage. **b.** The magnetoresistance also drops with increasing bias, indicating a bias-induced suppression of the spin-dependent transport mechanism. These trends are consistent with the notion that the junction barrier at the Fe_3O_4 interface is lowered at higher voltages, leading to more effective conduction pathways. Such voltage-dependent behavior can occur in variable-range hopping (VRH) as discussed in the main text, where the linear decrease in resistance suggests a significant reduction of the barrier.

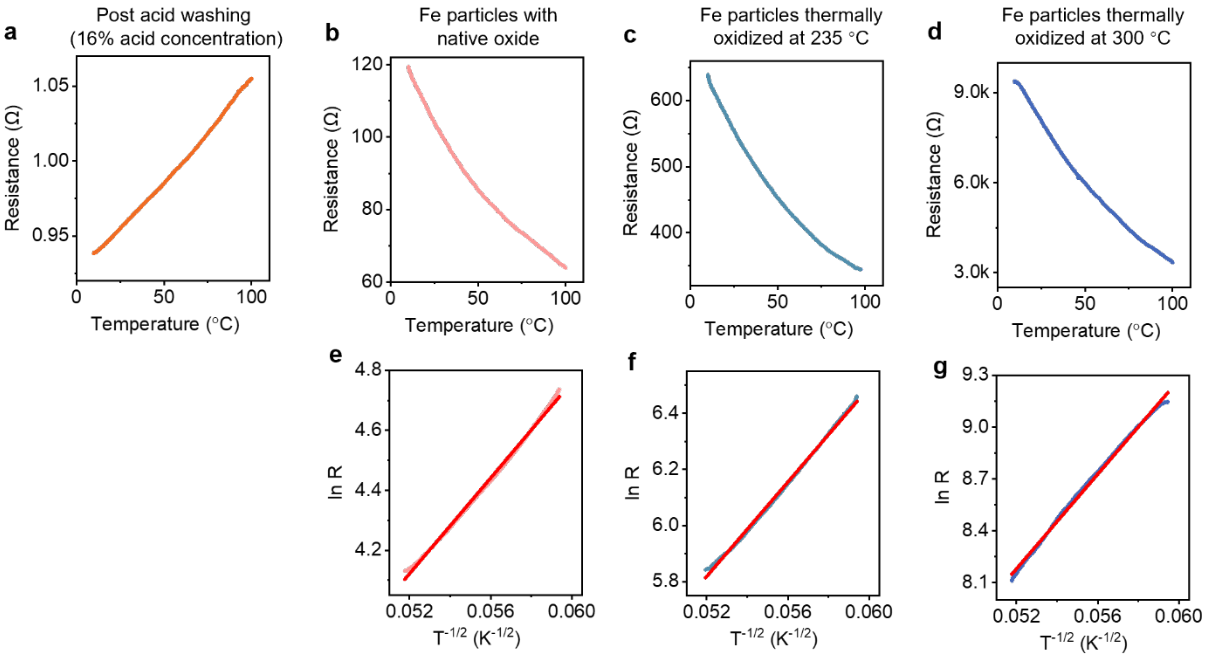

Supplementary Fig. 11. a-d. Temperature-dependent resistance variation of different samples, **e-g.** logarithmic plot of resistance as a function of $T^{-1/2}$. All datasets demonstrate consistent alignment with the variable-range hopping (VRH) conduction model (red line). Sample nomenclature used in the figures is the following **a.** printed sample treated by post acid washing (16% acid concentration). **b, e.** sample printed with commercial Fe particles with native oxide. **c, f.** sample printed with Fe particles thermally oxidized at 235°C. **d, g.** sample printed by Fe particles thermally oxidized at 300°C. The thermal oxidation was performed in 0.5 mbar vacuum condition.

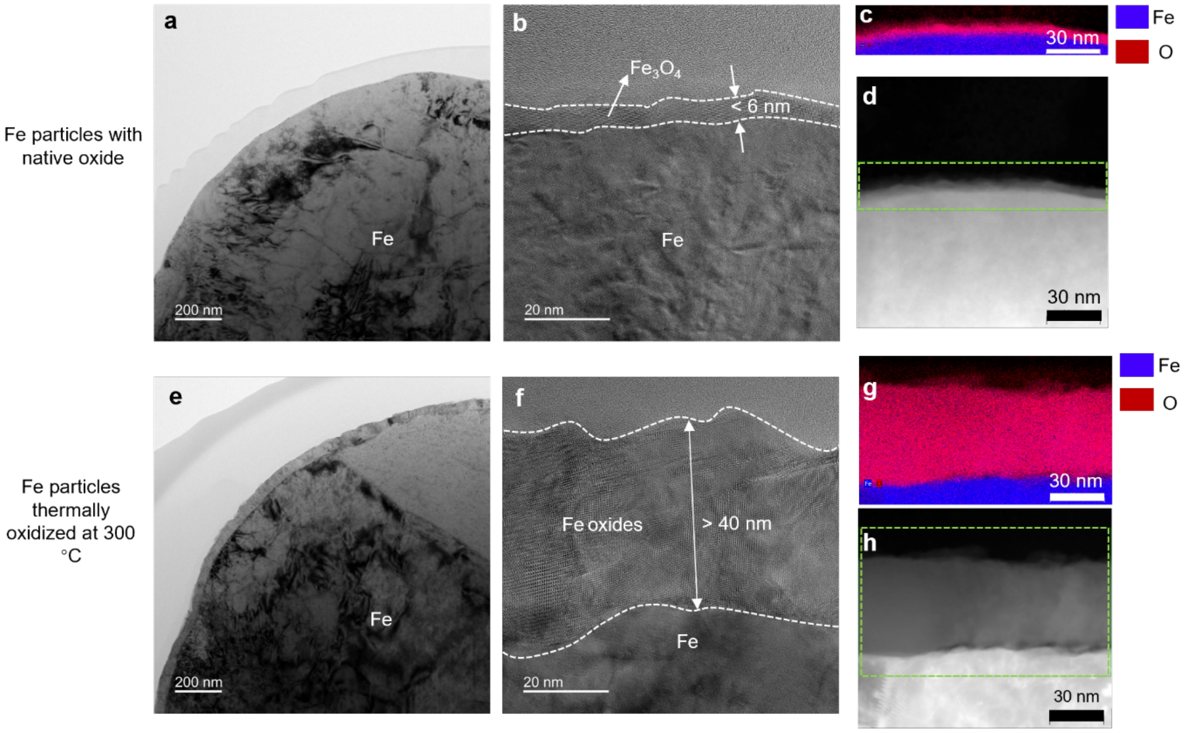

Supplementary Fig. 12. Cross-sectional TEM-based characterization of Fe/Fe_3O_4 core-shell microparticles with native oxide (a-d) and thermally oxidized at 300°C under 0.5 mbar vacuum for 30 min (e-h). a, e. Bright-field TEM images. **b, f.** HR-TEM images. **d, h**. HAADF-STEM images and **c, g** EDXS-based element distribution maps for the regions marked in panels **d** and **h**. According to XRD and Raman spectra analysis, the native oxide shell is composed of Fe_3O_4, while the shell of the thermally oxidized particles at 300°C is composed of Fe_3O_4 and α-Fe_2O_3.

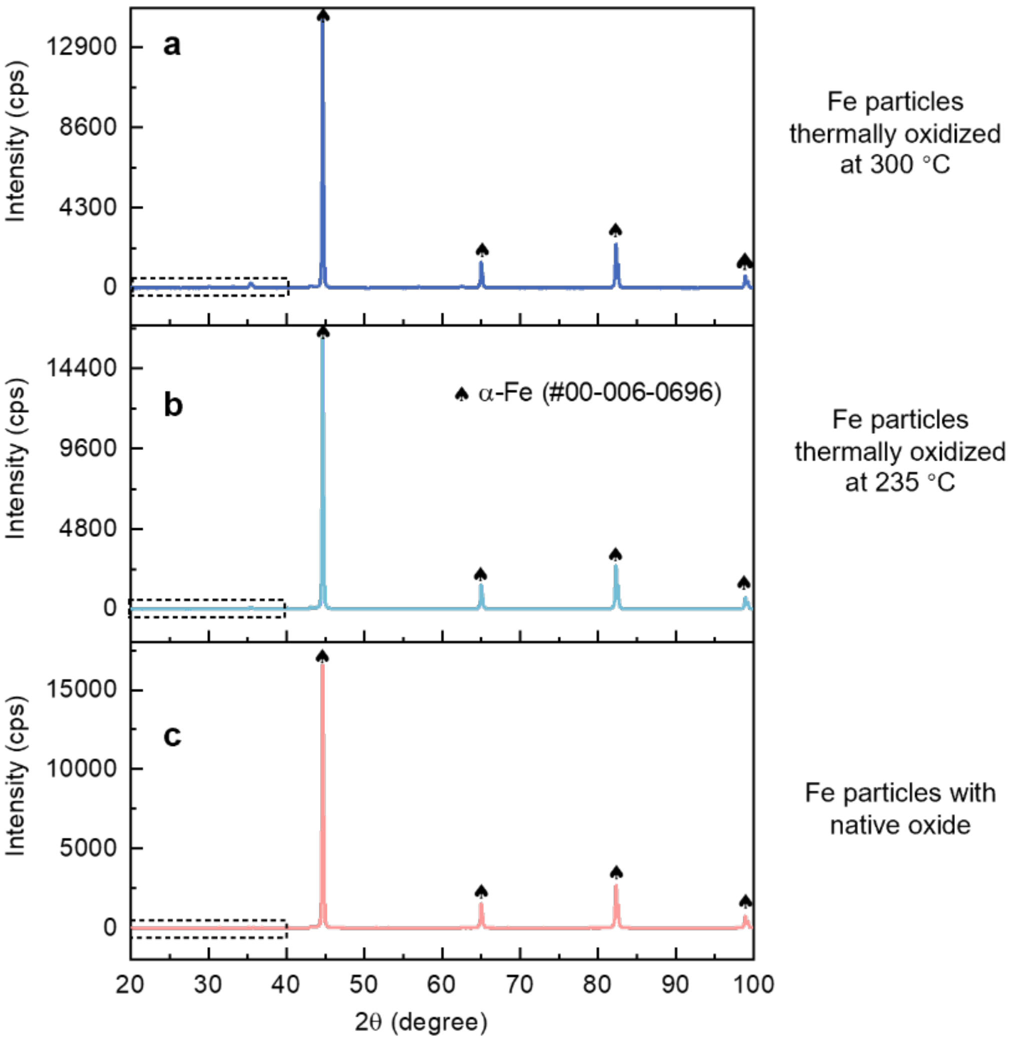

Supplementary Fig. 13. XRD analysis of Fe particles thermally oxidized at different temperatures under 0.5 mbar vacuum; a, XRD patterns of Fe particles thermally oxidized at 300°C. **b,** XRD patterns of Fe particles thermally oxidized at 235°C. **c.** XRD patterns of Fe particles with native oxide. Notably, the high-intensity diffraction peaks of metallic Fe overshadow the Fe_3O_4 peaks, suppressing their visibility. Fe_3O_4 diffraction peaks emerge at lower 2θ angles, as highlighted in zoomed-in views of the 2θ range between 20° and 40° (Fig. 3g in the main text). All thermal oxidation processes were conducted under a reduced pressure of 0.5 mbar.

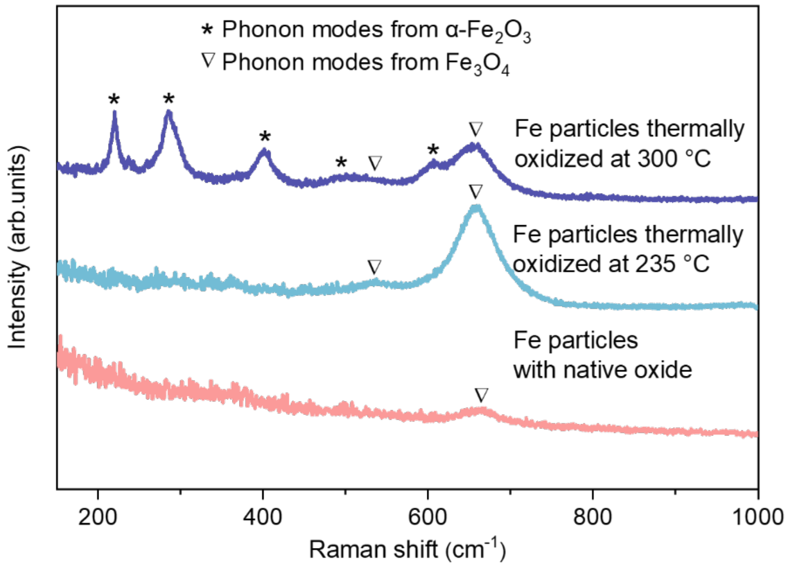

Supplementary Fig. 14. Room temperature Raman spectra of differently treated Fe particles. Green curve. Fe particles thermally oxidized at 300°C. **Blue curve.** Fe particles thermally oxidized at 235°C. **Red curve.** Fe particles with native oxide. The thermal oxidation was performed under 0.5 mbar vacuum conditions.

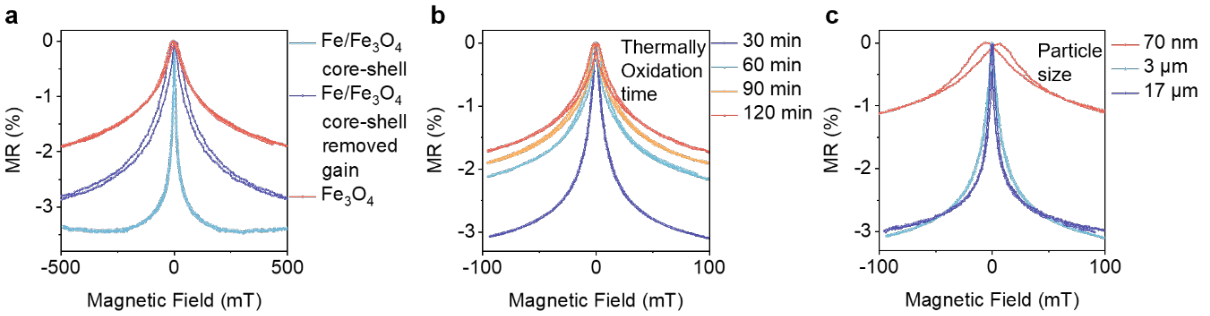

Supplementary Fig. 15. MR curve comparison. a, MR curves of printed Fe/Fe_3O_4 core-shell particles (cyan curve), with which removed 10 gain factor (purple curve) and printed Fe_3O_4 particles (red curve). **b,** MR curves of printed Fe/Fe_3O_4 core-shell particles with different time (30 min, 60 min, 90 min, and 120 min). **c,** MR curves of printed Fe/Fe_3O_4 core-shell particles with different size.

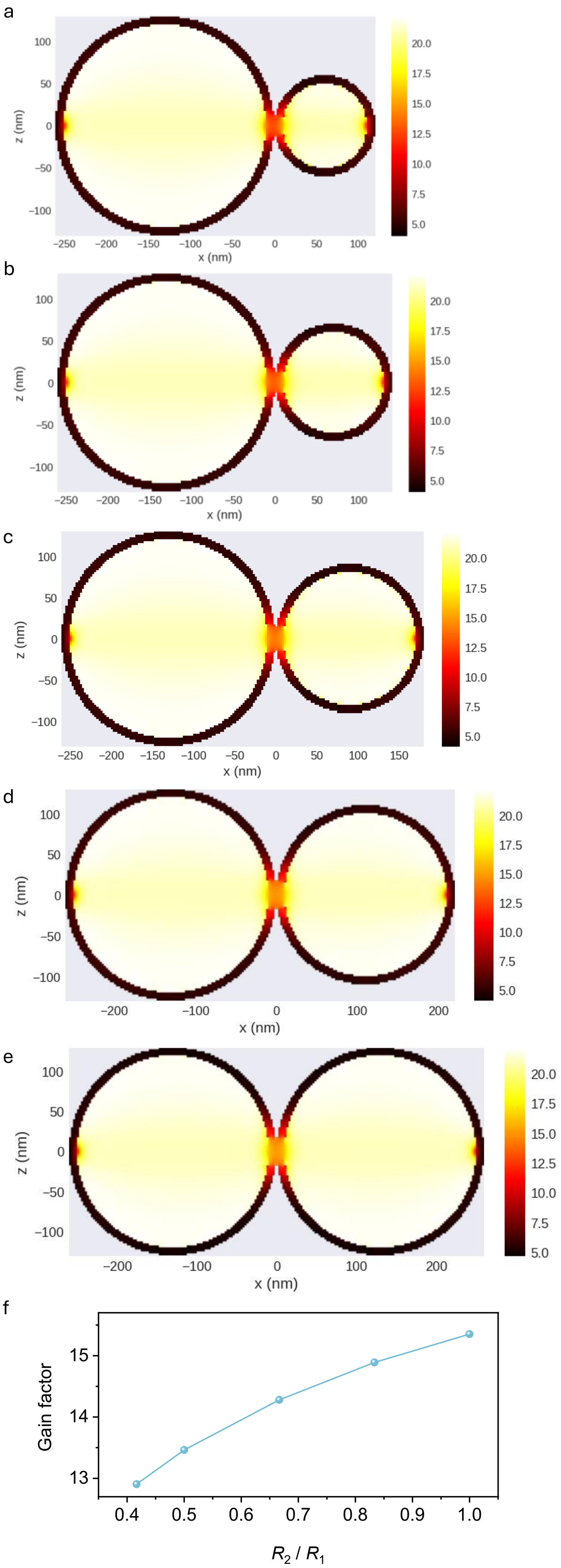

a
b
c
d
e
f
z (nm)
x (nm)
100
50
0
−50
−100
−250
−200
−150
−100
−50
0
50
100
150
200
20.0
17.5
15.0
12.5
10.0
7.5
5.0
Gain factor
15
14
13
0.4
0.5
0.6
0.7
0.8
0.9
1.0
R2 / R1

Supplementary Fig. 16. Simulation of particle-size-mismatch effects on the local gain factor at a core-shell junction. a-e. Spatial maps of the gain factor (colormap) in the axial cross-section of two contacting spherical Fe/Fe_3O_4 core–shell particles with different core-radius ratios. The larger particle is kept with a constant core radius, R_1, of 120 nm, while the smaller particle has core radii, R_2, of 50 nm (**a**), 60 nm (**b**), 80 nm (**c**), 100 nm (**d**), and 120 nm (**e**). In all cases, the Fe_3O_4 shell thickness is fixed at 10 nm. (**f**) Gain factor evaluated at the junction as a function of the core-radius ratio (R_2/R_1), showing only a modest variation across the investigated size-mismatch range.

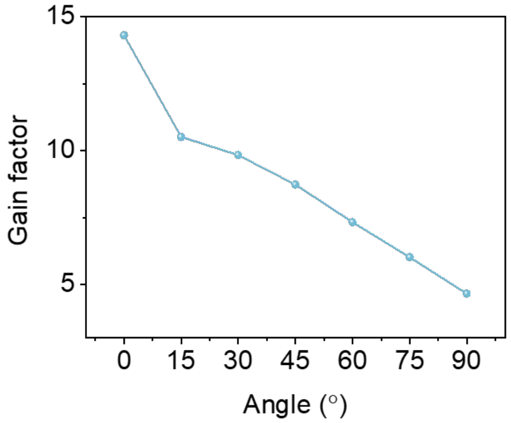

Supplementary Fig. 17. Gain factor as a function of the field direction for the pair of magnetic spheres with the fixed core diameter 240 nm and shell thickness of 10 nm. Direction of the external field of 100 mT is varied from the axis of symmetry of the system (0°) to be parallel to the mirror plane between spheres (90°). The gain factor is calculated for the average B-field at the junction between spheres.

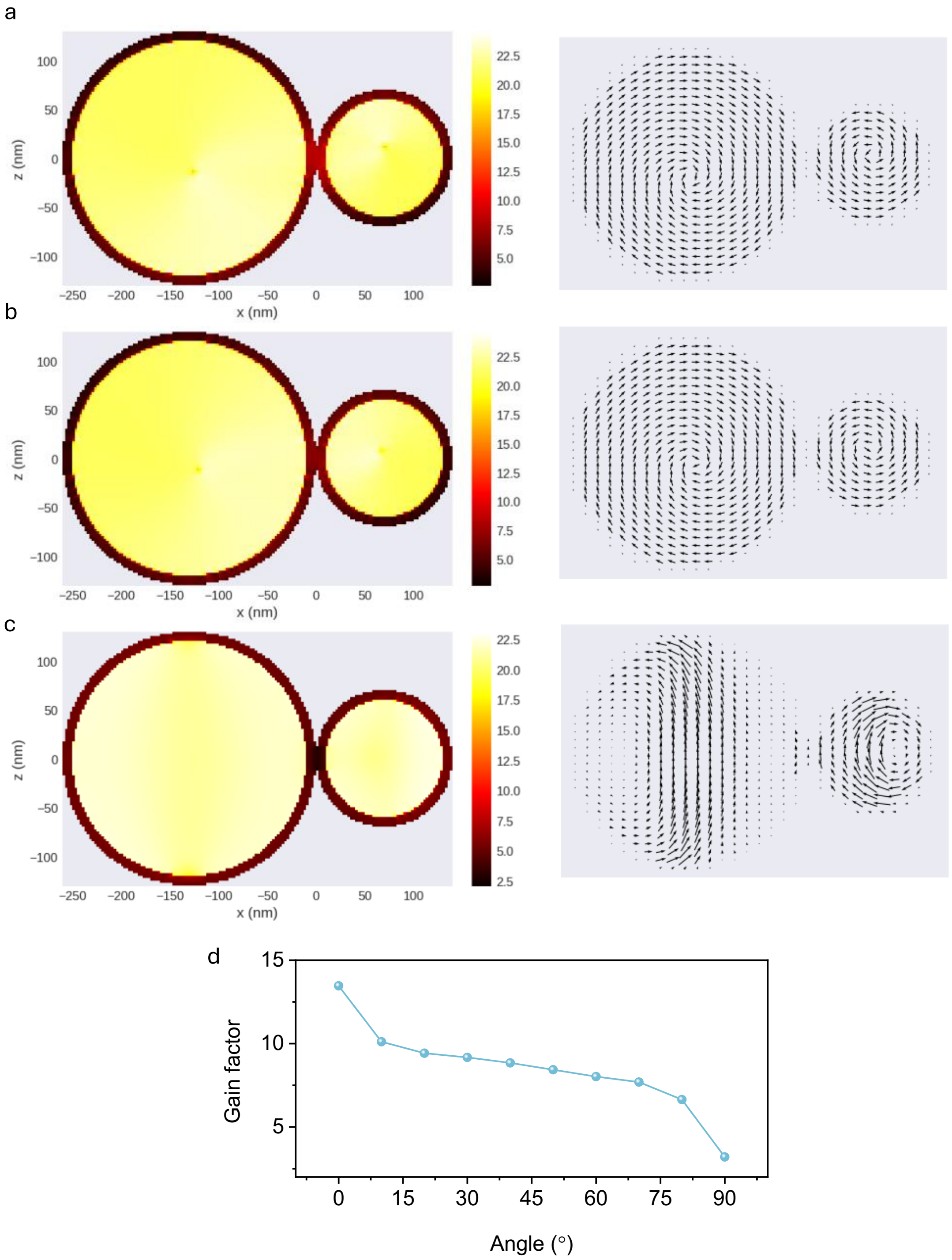

Supplementary Fig. 18. Simulation of field-orientation dependence of the local gain factor in a size-mismatched Fe/Fe_3O_4 particle pair. a-c. Spatial maps of the local gain factor (colormap) in the *x–z* axial cross-section of two contacting spherical core-shell Fe/Fe_3O_4 particles (core radii: 120 nm and 60 nm; shell thickness: 10 nm), together with the corresponding equilibrium magnetization distribution (arrows). An external magnetic field of 100 mT is applied with a tilt angle θ = 30° (panel **a**), 60° (panel **b**), and 90° (panel **c**) relative to the horizontal/interparticle axis. **d.** Gain factor at the junction as a function of θ, showing a progressive reduction with increasing tilt, consistent with the evolution of the equilibrium magnetic state near the junction.

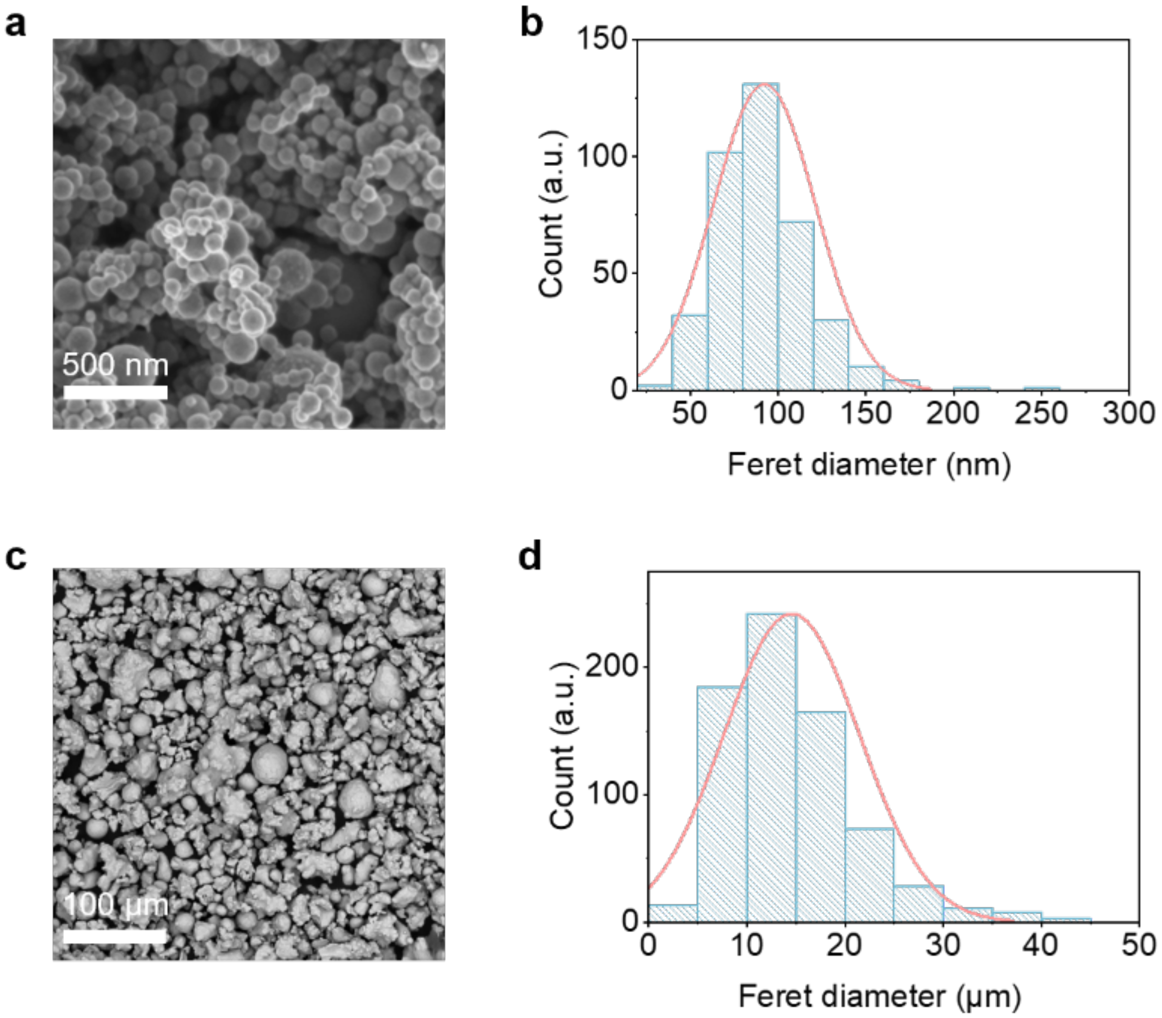

Supplementary Fig. 19. Distribution of Fe/Fe_3O_4 core-shell particle diameters. a, SEM image of Fe/Fe_3O_4 core-shell nano-particles. Scale bar: 500 nm. **b,** Distribution of nano-particle diameters. The average diameter is 90 nm. **c,** SEM image of Fe/Fe_3O_4 core-shell larger micro-particles. Scale bar: 100 μm. **d,** Distribution of micro-particle diameters. The average diameter is 15 μm.

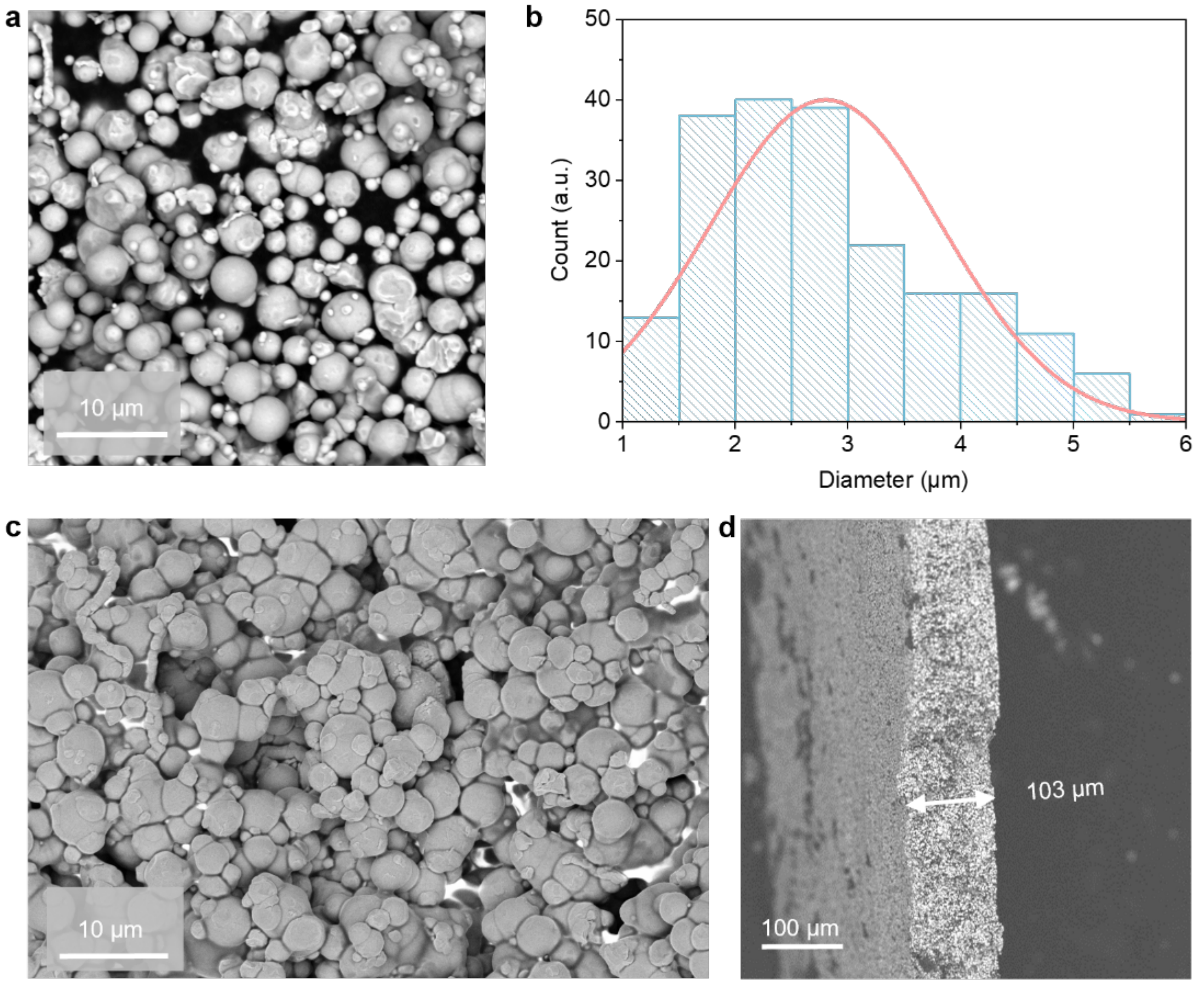

Supplementary Fig. 20. SEM characterization of Fe/Fe_3O_4 core-shell powders and printed sensor. **a,** SEM image of Fe/Fe_3O_4 core- shell microparticles. Scale bar: 10 μm. **b,** Distribution of microparticle diameters. The average diameter is 2.8 μm with spherical shape. **c,** Top view SEM image of printed Fe/Fe_3O_4-NaCMC sensor. Scale bar, 10 μm. **d,** Cross-section view SEM image of printed Fe/Fe_3O_4-NaCMC sensor. Scale bar, 100 μm. The thickness of printed sensor is about 100 μm.

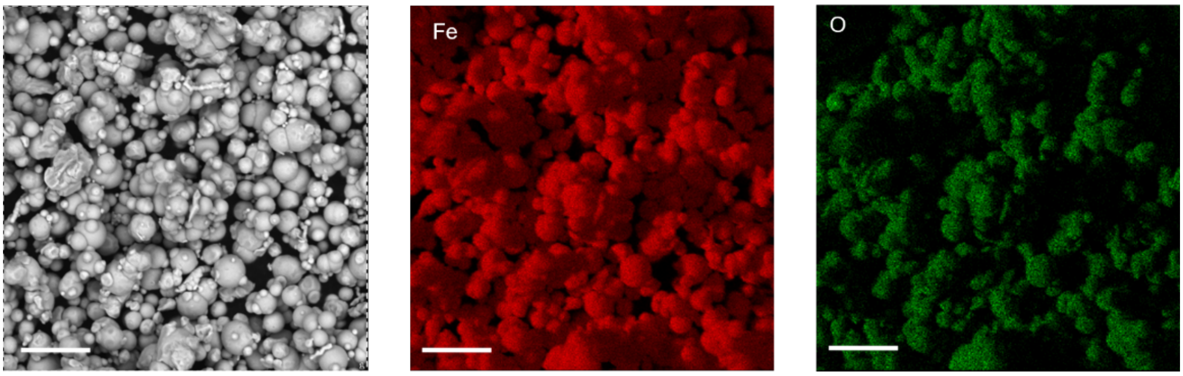

Supplementary Fig. 21. Energy-dispersive X-ray Spectroscopy (EDX) maps of Fe/Fe_3O_4 core-shell microparticles. O signal from the oxide shell. Scale bars, 10 µm.

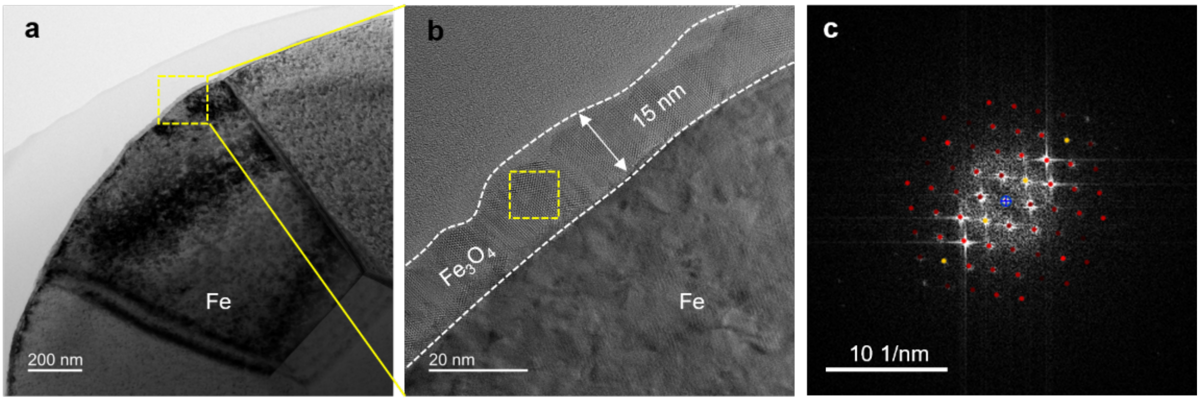

Supplementary Fig. 22. Cross-sectional TEM characterization of an Fe/Fe_3O_4 core-shell microparticle thermally oxidized at 235°C under 0.5 mbar vacuum for 30 min. a, Bright-field TEM image. **b,** HR-TEM image for the region marked in panel (a). **c.** Fast Fourier transform of the region marked in panel (b) superimposed with a simulated diffraction pattern based on the magnetite structure in [101] zone axis geometry.

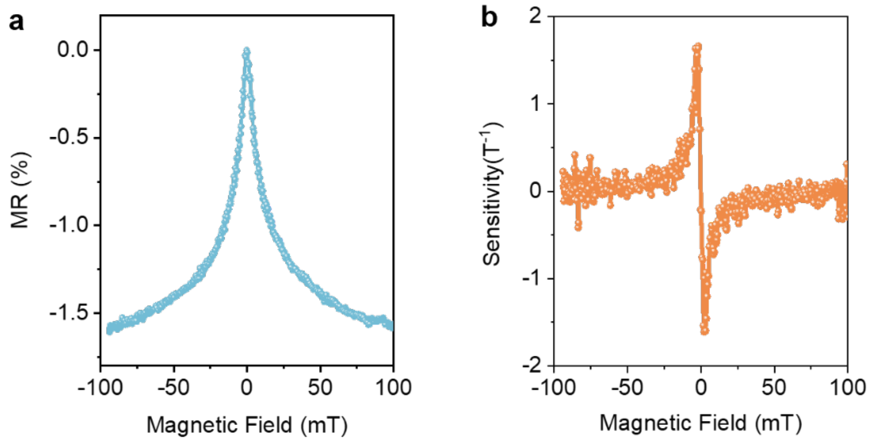

Supplementary Fig. 23. Characterization of printed MR sensors fabricated with the Fe particles thermally oxidized at 235°C under ambient condition (non-vacuum, 30 min, 235°C). **a,** Magnetoresistance and **b,** magnetoresistive sensitivity of the sensors. The uncontrollable thermal oxidation at ambient condition is difficult to tailor the high crystalline quality of Fe_3O_4 shell and limited MR performance.

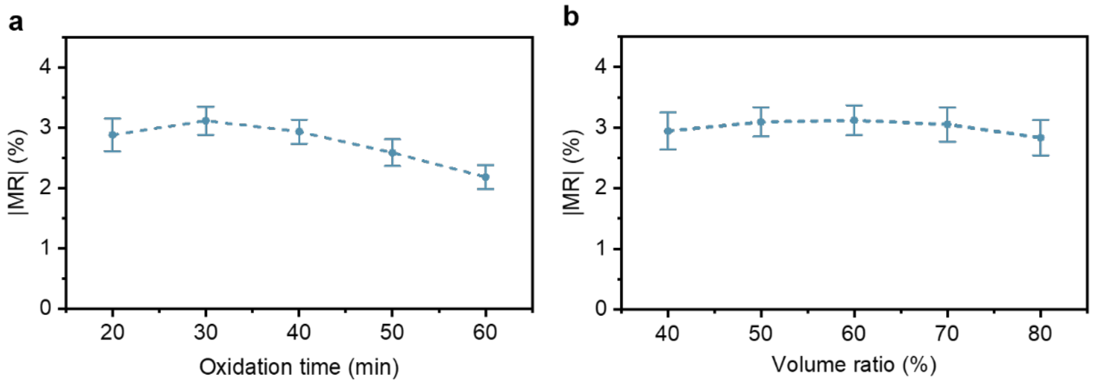

Supplementary Fig. 24. a. MR performance of printed sensors with the Fe particles as a function of oxidation time (235°C under 0.5 mbar vacuum). Error bars are presented as mean ± SD. **b.** MR performance of printed sensors with different particle volume ratio in the NaCMC matrix (particles oxidized at 235°C for 30 min under 0.5 mbar vacuum). Error bars are presented as mean ± SD.

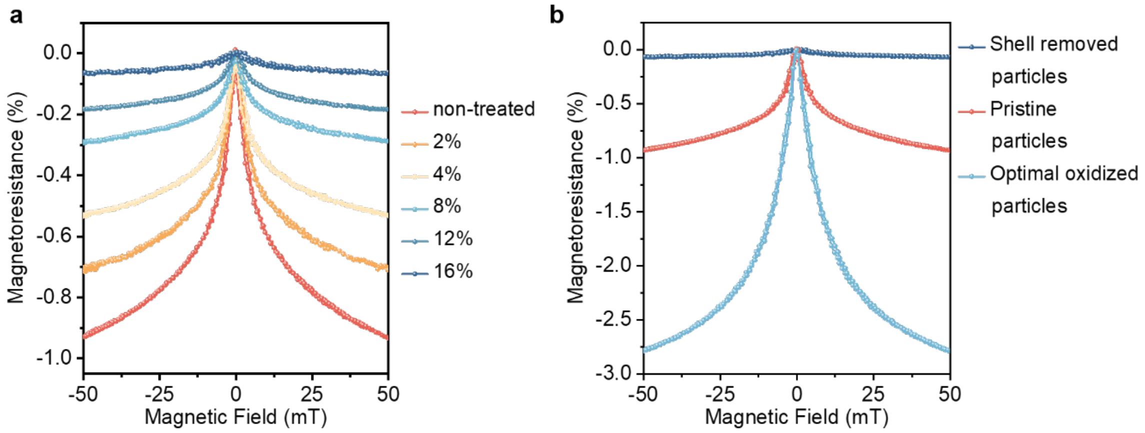

Supplementary Fig. 25. a. MR curves of printed Fe/Fe_3O_4 sensor post-treated by vitamin C solution with different concentration. **b.** MR curves of printed Fe/Fe_3O_4 sensor with optimally oxidized particles, pristine particles (naturally oxidized) and shell removed particles.

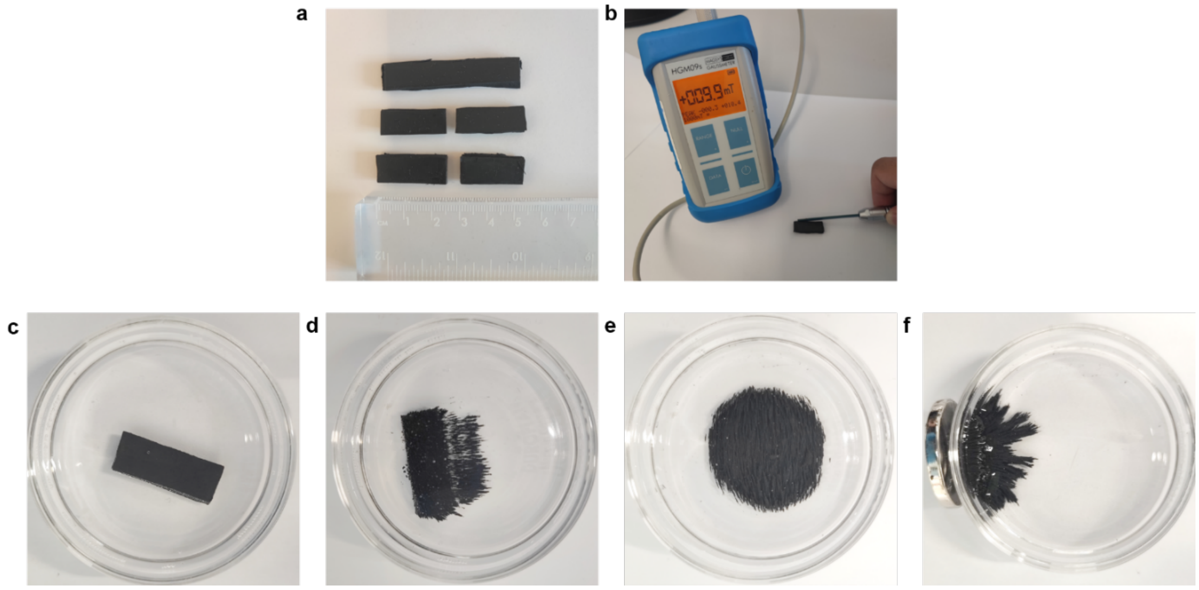

Supplementary Fig. 26. a, Photo of sustainable printed magnets. **b,** Depiction of the magnetic field produced by these printed magnets. **c, d, and e,** the degradation of the printed magnet in warm water (70°C). **f,** Dispersed ferrite powders were recycled by a permanent magnet. We used these magnets for smart box applications in the main text. These sustainable magnets consist of a biocompatible composite comprising 70 wt% strontium ferrite powder and 30 wt% gelatin-glycerol gel. The non-volatile nature of glycerol ensures long-term stability of the gel, while the fabrication process adheres to eco-friendly principles, avoiding hazardous chemicals. However, the relatively low magnetic performance of the sustainable strontium ferrite powder restricts the strength of the generated magnetic field. This limitation underscores the need for highly sensitive biodegradable MR sensors to advance sustainable and fully biodegradable electronic systems.

References

1. Karnaushenko, D., Makarov, D., Yan, C., Streubel, R. & Schmidt, O. G. Printable giant magnetoresistive devices. *Adv. Mater.* **24**, 4518–4522 (2012).

2. Karnaushenko, D. *et al.* High-performance magnetic sensorics for printable and flexible electronics. *Adv. Mater* **27**, 880–885 (2015).

3. Ha, M. *et al.* Printable and Stretchable Giant Magnetoresistive Sensors for Highly Compliant and Skin-Conformal Electronics. *Adv. Mater* **33**, 2005521 (2021).

4. Gupta, P. *et al.* Large Scale Exchange Coupled Metallic Multilayers by Roll-to-Roll (R2R) Process for Advanced Printed Magnetoelectronics. *Adv. Mater. Technol* **7**, 2200190 (2022).

5. Wang, X. *et al.* Printed magnetoresistive sensors for recyclable magnetoelectronics. *J. Mater. Chem. A* **12**, 24906-24915 (2024).

6. Cox, B., Davis, D. & Crews, N. Creating magnetic field sensors from GMR nanowire networks. *Sens. Actuators, A* **203**, 335–340 (2013).

7. Sergio, E. *et al.* Printable anisotropic magnetoresistance sensors for highly compliant electronics. *Appl. Phys. A* **127**, 280 (2021).

8. Xu, R. *et al.* Self-healable printed magnetic field sensors using alternating magnetic fields. *Nat. Commun.* **13**, 6587 (2022).

9. Xu, R. *et al.* Printed Conformal and Transparent Magnetoresistive Sensors for Seamless Integration and Environment-Resilient Touchless Interaction. *ACS Nano* **19**, 21891–21903 (2025).

10. Oliveros-Mata, E. S. *et al.* Dispenser Printed Bismuth-Based Magnetic Field Sensors with Non-Saturating Large Magnetoresistance for Touchless Interactive Surfaces. *Adv. Mater. Technol.* **7**, 2200227 (2022).

11. Dugay, J. *et al.* Room-Temperature Tunnel Magnetoresistance in Self-Assembled Chemically Synthesized Metallic Iron Nanoparticles. *Nano Lett.* **11**, 5128–5134 (2011).

12. Usmani, S. *et al.* Tunnel magnetoresistance and cotunneling in assemblies of chemically synthesized FeCo nanoparticles. *Phys. Rev. B* **98**, 104433 (2018).

13. Tondra, M. *et al.* Thickness dependence of the anisotropic magnetoresistance in epitaxial iron films. *J. Appl. Phys.* **73**, 6393–6395 (1993).

14. Coleman, R. V. & Isin, A. Magnetoresistance in iron single crystals. *J. Appl. Phys* **37**, 1028–1029 (1966).

15. Isin, A & Coleman, R. V. Temperature Dependence of Magnetoresistance in Iron. *Phys. Rev.* **142**, 372 (1966).

16. Martinez-Boubeta, C. *et al.* Temperature dependence of the magnetoresistance in Fe/MgO core/shell nanoparticles. *Appl. Phys. Lett.* **94**, 2–5 (2009).

17. Guo, Z. *et al.* Giant magnetoresistance behavior of an iron/carbonized polyurethane nanocomposite. *Appl. Phys. Lett.* **90**, 053111 (2007).

18. Guo, Z., Hahn, H. T., Lin, H., Karki, A. B. & Young, D. P. Magnetic and magnetoresistance behaviors of particulate iron/vinyl ester resin nanocomposites. *J. Appl. Phys.* **104**, 014314 (2008).

19. Zhang, D. *et al.* Magnetic and magnetoresistance behaviors of solvent extracted particulate iron/polyacrylonitrile nanocomposites. *J. Phys. Chem. C* **114**, 212–219 (2010).

20. Mi, W. B., Shen, J. J., Jiang, E. Y. & Bai, H. L. Microstructure, magnetic and magneto-

transport properties of polycrystalline Fe3O4 films. *Acta Mater.* **55**, 1919–1926 (2007).

21. Liu, H. *et al.* Large room-temperature spin-dependent tunneling magnetoresistance in polycrystalline Fe3O4 films. *Appl. Phys. Lett.* **83**, 3531–3533 (2003).

22. Furubayashi, T. Magnetoresistance of magnetite films prepared by reactive evaporation. *J. Appl. Phys.* **93**, 8026–8028 (2003).

23. Venkatesan, M., Nawka, S., Pillai, S. C. & Coey, J. M. D. Enhanced magnetoresistance in nanocrystalline magnetite. *J. Appl. Phys.* **8023**, 2001–2004 (2003).

24. Kostopoulos, D. Magnetoresistance of magnetite. *Appl. Phys. Lett.* **9**, 523–527 (1998).

25. Yue, F. J. *et al.* Large low-field magnetoresistance in Fe3O4/molecule nanoparticles at room temperature. *J. Phys. D:Appl. Phys.* **44**, 025001 (2011).

26. Wang, S. *et al.* Room-temperature spin-dependent tunneling through molecules. *Appl. Phys. Lett.* **98**, 2009–2012 (2011).

27. Lv, Z. P. *et al.* Controlling the assembly and spin transport of tetrathiafulvalene carboxylate coated iron oxide nanoparticles. *J. Mater. Chem. C* **5**, 7200–7206 (2017).

28. Lv, Z. P. *et al.* Tuning Electron-Conduction and Spin Transport in Magnetic Iron Oxide Nanoparticle Assemblies via Tetrathiafulvalene-Fused Ligands. *ACS Nano* **9**, 12205–12213 (2015).

29. Wang, T. *et al.* Enhancing low-field magnetoresistance in magnetite nanoparticles: Via zinc substitution. *Phys. Chem. Chem. Phys.* **20**, 17245–17252 (2018).
