# Supplementary material for: Eco-sustainable magnetoresistive sensors towards disposable magnetoelectronics
Source: Nat Commun. 2026 Mar 27;17:3034. doi: 10.1038/s41467-026-71077-9 (PMC13036069; doi:10.1038/s41467-026-71077-9)
Supplement: Supplementary file 2 — Description of Additional Supplementary Files [file 41467_2026_71077_MOESM2_ESM.pdf]

Description of Additional Supplementary Files

Supplementary Movie 1. Large scale fabrication by screen printing.

Supplementary Movie 2. Recycling dispersed Fe/Fe_3O_4 core-shell particles from water by permanent magnet.

Supplementary Movie 3. Separating and recycling dispersed Fe/Fe_3O_4 core-shell particles from mixed powders.

Supplementary Movie 4. Human-machine interactive application of disposable printed Fe/Fe_3O_4-NaCMC sensors.
